# Crystal structure of nitrido[5,10,15,20-tetra­kis(4-methylphenyl)­porphyrinato]­manganese(V)

**DOI:** 10.1107/S1600536814020558

**Published:** 2014-09-24

**Authors:** Mason R. Shields, Ilia A. Guzei, James G. Goll

**Affiliations:** a Department of Chemistry, Geoscience, and Physics, 1000 Edgewood College Drive, Edgewood College, Madison, WI 53711, USA; b Department of Chemistry, University of Wisconsin-Madison, 1101 University Ave, Madison, WI 53706, USA

**Keywords:** porphyrin, nitride, crystal structure, manganese(V) complex

## Abstract

The title compound, [Mn(C_48_H_36_N_4_)(N)], is a manganese(V) complex with the transition metal in a square-pyramidal coordination geometry and a nitride as the axial ligand. The complex resides on a crystallographic inversion center and only one half of it is symmetry independent. The Mn^V^ atom and the nitride N atom are equally disordered across the inversion center. The Mn N distance is 1.516 (4) Å. The Mn^V^ atom is displaced from the plane defined by the four equatorial nitro­gen atoms toward the nitride ligand by 0.3162 (6) Å.

## Abstract 2

In the title compound the Mn N_nitride_ distance is 1.516 (4) Å. The Mn atom is displaced from the plane defined by the four equatorial nitro­gen atoms toward the nitride ligand by 0.3162 (6) Å.

## Chemical context   

Tetra­pyrrole ligands have been used as a supporting ligand to stabilize high-valent, manganese compounds with manganese in 5-coordination and nitride ligands with short Mn N bond lengths. These complexes are characterized by Mn N distances of approximately 1.5 Å and the central metal displaced from the plane of the four equatorial N atoms toward the nitride ligand by up to 0.55 Å. In the course of our studies of Mn complexes we prepared and isolated the title complex, 5,10,15,20-tetra­kis-tetra­tolyl­porphyrinato­nitrido­manganese(V) (I), and con­duc­ted its structural characterization to investigate how its geometry compares to that of its congeners.
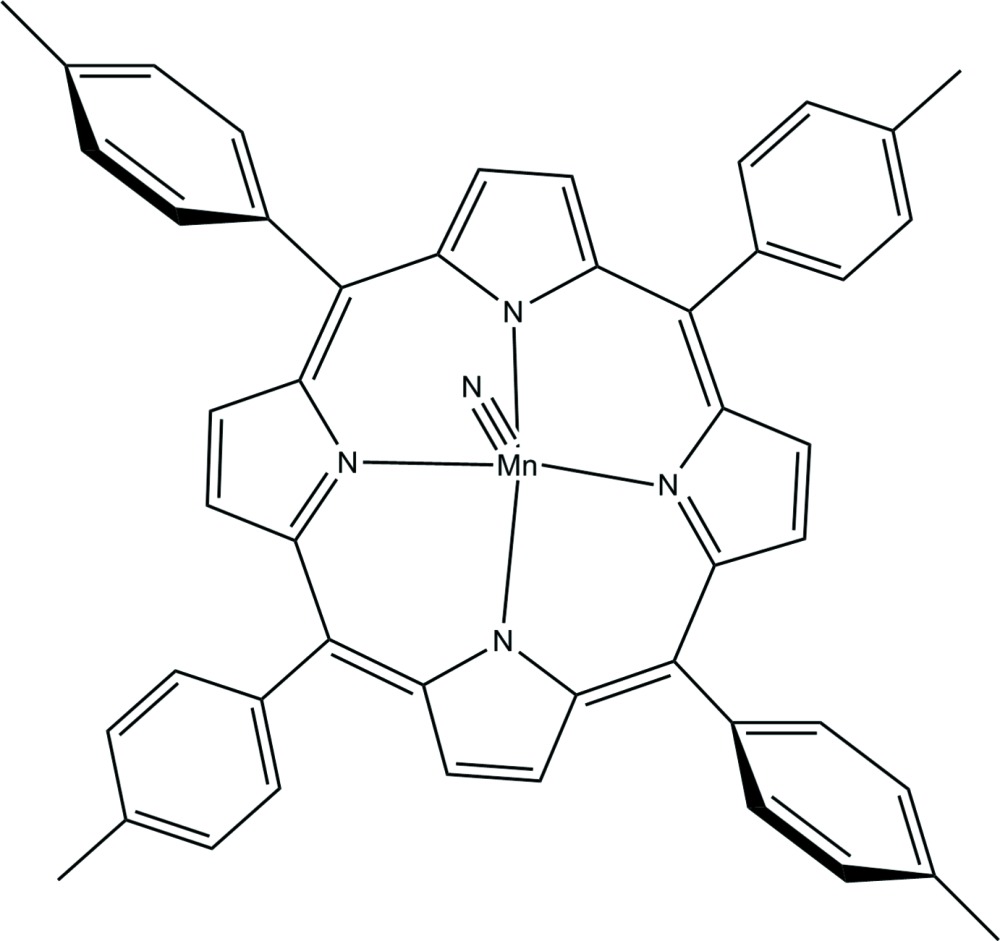


We have found five examples of five-coordinate nitride Mn complexes deposited with the Cambridge Structural Database (CSD; Allen, 2002 ▶): (tetra­kis-tetra-4-meth­oxy­phen­yl)porphyrinato­nitrido­manganese(V) (II) (Hill & Hollander, 1982 ▶), (5,15-dimethyl-2,3,7,8,12,13,17,18-octa­ethyl-5*H*,15*H*-porphinato)nitridomanganese(V) (III) (Buchler *et al.*, 1983 ▶),(5,10,15-tris­(penta­fluoro­phen­yl)corrole)(mesityl­imido)manganese(V) toluene solvate (IV) (Eikey *et al.*, 2002 ▶), (2,3,7,8,12,13,17,18-octa­kis­(4-*t*-butyl­phen­yl)corrolazinato)-(mesitylimido)-manganese(V) di­chloro­methane solvate (V) (Lansky *et al.*, 2006 ▶), and nitrido-(6,11,17-tris­(4-nitro­phen­yl)-16,21,22,23,24-pentaaza­penta­cyclo­[16.2.1.12,5.17,10.112,15]tetra­cosa-1,3,5,7,9,11,13,15,17,19-deca­enato)manganese(V) di­chloro­methane solv­ate, (VI) (Singh *et al.*, 2013 ▶). Herein we report the comparison of key structural parameters of (I) to those of (II)–(VI).

## Structural commentary   

**Figure 1:**
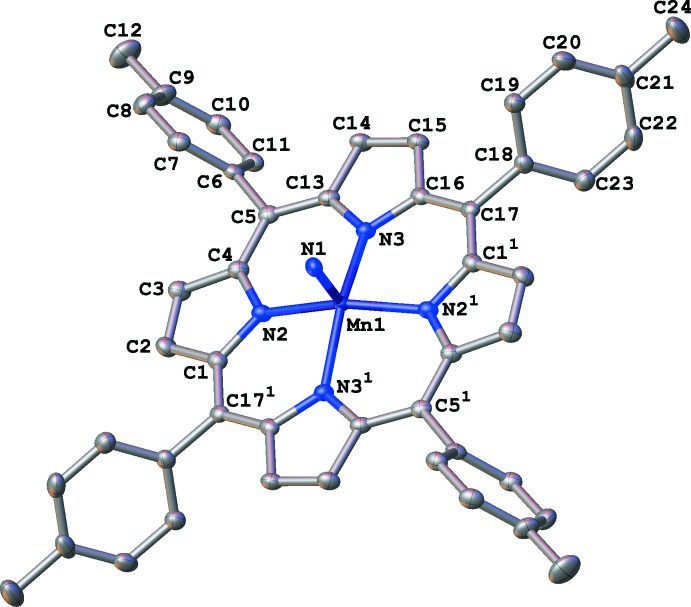
A mol­ecular drawing of (I) shown with displacement parameters at the 50% probability level. All H atoms and the disordered mates of atoms Mn1 and N1 are omitted. [Symmetry operator (1): −*x* + 1, −*y* + 1, −*z*.]

In the crystal structure of the title complex (I) (Fig. 1 ▶), the central Mn^V^ atom possesses a square-pyramidal geometry. The equatorial plane is formed by the four nitro­gen atoms of the porphyrin whereas the apical position is occupied by the nitride ligand. The complex resides on a crystallographic inversion center and only one half of it is symmetry independent. The Mn1 atom and nitride ligand atom N1 are equally disordered over two positions. This crystallographic behavior (disorder about an inversion center) was also observed in the case of (II). Whereas both complexes exhibit inversion symmetry, the Mn—N distances in them are not equal pairwise (as one would expect based on the fact that only one half of the complex is unique) because the Mn^V^ atom is displaced from the equatorial plane not perpendicularly to it but at a small angle. Thus, the Mn—N distances in (I) range from 1.958 (2) to 2.070 (2) Å and between 1.983 (2) and 2.060 (2) Å in (II). The selected geometrical parameters for (I)–(VI) are presented in Table 1 ▶. A somewhat counter-intuitive trend correlates the average Mn—N(eq) distance and the displacement of the Mn from the equatorial plane: the shorter the Mn—N(eq) distance, the larger the displacement. The correlation between the Mn—N(eq) distances and Mn N distance is not consistent, but in general the shorter the Mn—N(eq) distances, the longer the Mn N bond length, as might be expected. We have also conducted a CSD search for Mn^V^ complexes with manganese in six-coordination and with a nitride ligand and found seven relevant compounds, but none of them was a porphyrin or a porphyrin derivative. The intention was to determine whether the expected metal–ligand bond lengthening occurs as the metal coordination number increases. It was found that for the five-coordinate (I)–(VI) the average Mn N distance is 1.54 (5) Å, whereas for the seven six-coordinate complexes this distance is 1.527 (10) Å. Thus, the difference in the nature of the ligands (porphyrin *vs* tetra-aza­cyclo-tetra­deca­ne) accounts for the prediction ‘reversal’.

## Supra­molecular features   

**Figure 2:**
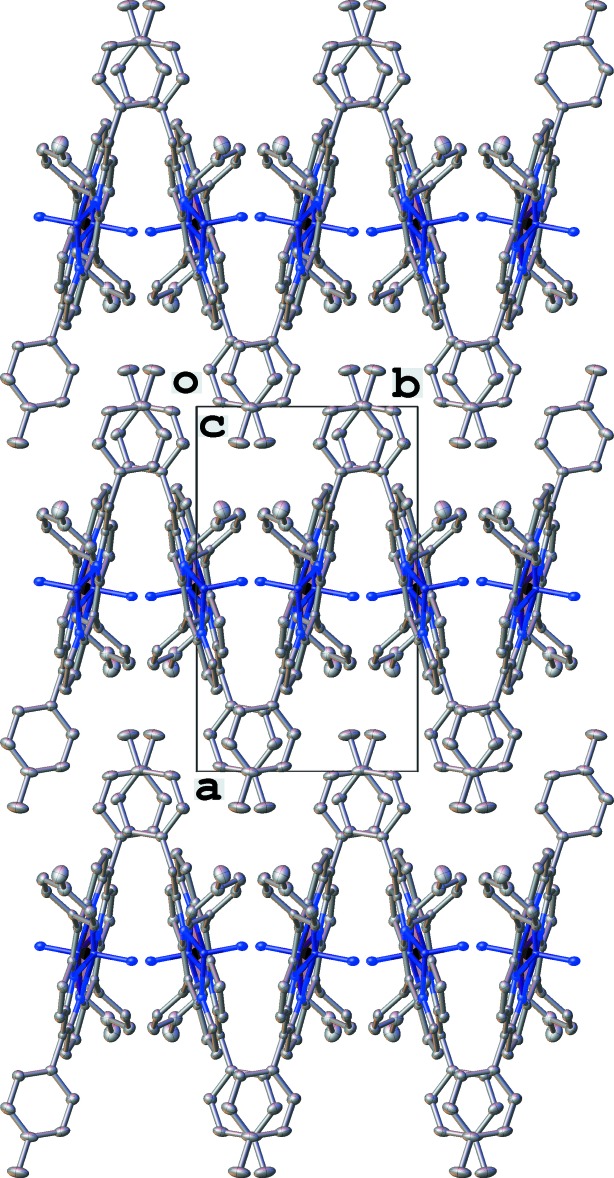
A packing diagram of (I) shown along the [001] direction. All H atoms are omitted.

Whereas there are possible weak non-classical inter­actions such as C—H⋯π and C—H⋯N(nitride) (Table 2 ▶), no π–π stacking inter­actions are detected. The mol­ecules pack forming porphyrin/tolyl layers along the [100] direction with a 14.2619 (10) Å separation between identical layers (Fig. 2 ▶). The dihedral angle between the adjacent porphyrin core planes within the same layer is 30.037 (4)°.

## Synthesis and crystallization   

The title compound, 5,10,15,20-tetra­kis-tetra­tolyl­porphyrin­ato­nitridomanganese(V), was prepared according to the procedure developed by Buchler *et al.* (1982 ▶). (TTP)Mn(C_2_H_3_O_2_) where TTP is the dianion of *meso*-tetratolylporphyrin (2.08 g, 2.65 mmol) was dissolved in methanol and eluted down an alumina column with methanol. The methanol was removed and the product redissolved in 400 ml di­chloro­methane. This solution was treated with 12 ml of an ammonia solution made by diluting 2 ml of concentrated ammonia with 10 ml of water and allowed to stir for fifteen minutes. A 10% sodium hypochlorite solution (6 ml) was added and the reaction was stirred an additional 15 minutes, resulting in a red solution. The solution was then washed with two 100 ml portions of water to remove the excess ammonia and hypochlorite and the sodium chloride formed during the reaction. The filtrate was placed on a neutral alumina column and the product was eluted with di­chloro­methane. Unreacted manganese(III) porphyrin can be recovered by eluting with methanol. The product was dried under reduced pressure. UV–vis (λ_max_ 535, 421 nm) are in excellent agreement with those obtained by Buchler *et al.* (1982 ▶) (536 and 421 nm). The NMR spectrum (Anasazi 60 MHz FT–NMR: ^1^H NMR (296 K, CDCl_3_, p.p.m.) 8.94 (*s*, 8H), 8.03 (*d*, 8H), 7.53 (*d* 8H), 2.68 (*s*, 12H)) matches the literature data as well. A yield of 1.82 g, 93% based on (TTP)Mn(C_2_H_3_O_2_) was obtained. (TTP)Mn N used to grow the crystal for the structural determination was purified by taking a di­chloro­methane solution and eluting through neutral alumina column with di­chloro­methane.

## Refinement   

Crystal data, data collection and structure refinement details are summarized in Table 3 ▶. All hydrogen atoms were included in the structure-factor calculation at idealized positions and were allowed to ride on the neighboring atoms with relative isotropic displacement coefficients.

## Supplementary Material

Crystal structure: contains datablock(s) I. DOI: 10.1107/S1600536814020558/zl2598sup1.cif

Structure factors: contains datablock(s) I. DOI: 10.1107/S1600536814020558/zl2598Isup2.hkl

Supporting information file. DOI: 10.1107/S1600536814020558/zl2598Isup3.png

Supporting information file. DOI: 10.1107/S1600536814020558/zl2598Isup4.cdx

CCDC reference: 1024311

Additional supporting information:  crystallographic information; 3D view; checkCIF report

## Figures and Tables

**Table 1:** Selected metric parameters for (I)–(VI) (Å)

Compound	Mn N	Mn—N(eq, av)	Mn—N4 displacement
(I)	1.516 (4)	2.02 (5)	0.3162 (6)
(II)	1.512 (2)	2.02 (3)	0.388
(III)	1.512	2.006 (3)	0.426
(IV)	1.613	1.92 (2)	0.513
(V)	1.595	1.893 (10)	0.550
(VI)	1.512	1.99 (3)	0.460

**Table 2:** Hydrogen-bond geometry (Å, °) *Cg*1 and *Cg*2 are the centroids of the N3/C13–C16 and C6–C11 rings, respectively.

*D*—H⋯*A*	*D*—H	H⋯*A*	*D*⋯*A*	*D*—H⋯*A*
C10—H10⋯N1^i^	0.95	2.42	3.203 (5)	140
C11—H11⋯*Cg*1^ii^	0.95	2.77	3.332 (3)	119
C19—H19⋯*Cg*2^iii^	0.95	2.68	3.619 (3)	170

Symmetry codes: (i) 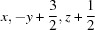; (ii) 
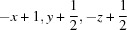
; (iii) 
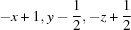
.

**Table 3:** Experimental details

Crystal data	
Chemical formula	[Mn(C_48_H_36_N_4_)(N)]
*M*_r_	737.76
Crystal system, space group	Monoclinic, *P*2_1_/*c*
Temperature (K)	100
*a*, *b*, *c* (Å)	14.2619 (10), 8.6200 (11), 15.4685 (18)
β (°)	94.188 (7)
*V* (Å^3^)	1896.6 (4)
*Z*	2
Radiation type	Cu *K*α
μ (mm^−1^)	3.14
Crystal size (mm)	0.17 × 0.11 × 0.03

Data collection	
Diffractometer	Bruker *SMART* *APEX2* area detector
Absorption correction	Multi-scan (*SADABS*; Bruker, 2012 ▶)
*T*_min_, *T*_max_	0.529, 0.662
No. of measured, independent and observed [*I* > 2σ(*I*)] reflections	30677, 3602, 3184
*R*_int_	0.050
(sin θ/λ)_max_ (Å^−1^)	0.610

Refinement	
*R*[*F* ^2^ > 2σ(*F* ^2^)], *wR*(*F* ^2^), *S*	0.051, 0.131, 1.03
No. of reflections	3602
No. of parameters	255
H-atom treatment	H-atom parameters constrained
Δρ_max_, Δρ_min_ (e Å^−3^)	0.33, −0.38

Computer programs: *APEX2* and *SAINT-Plus* (Bruker, 2014 ▶), *SHELXT* and *SHELXL* (Sheldrick, 2008 ▶), *OLEX2* (Dolomanov *et al.*, 2009 ▶), *publCIF* (Westrip, 2010 ▶) and *GX* (Guzei, 2013 ▶).

## supplementary crystallographic information

### Crystal data

**Table:**

[Mn(C_48_H_36_N_4_)(N)]	*F*(000) = 768
*M**_r_* = 737.76	*D*_x_ = 1.292 Mg m^−^^3^
Monoclinic, *P*2_1_/*c*	Cu *K*α radiation, λ = 1.54178 Å
*a* = 14.2619 (10) Å	Cell parameters from 6388 reflections
*b* = 8.6200 (11) Å	θ = 3.1–70.1°
*c* = 15.4685 (18) Å	µ = 3.14 mm^−^^1^
β = 94.188 (7)°	*T* = 100 K
*V* = 1896.6 (4) Å^3^	Plate, red
*Z* = 2	0.17 × 0.11 × 0.03 mm

### Data collection

**Table:**

Bruker SMART APEX2 area detector diffractometer	3602 independent reflections
Radiation source: sealed X-ray tube, Siemens, K FFCU 2K 90	3184 reflections with *I* > 2σ(*I*)
Equatorially mounted graphite monochromator	*R*_int_ = 0.050
Detector resolution: 7.9 pixels mm^-1^	θ_max_ = 70.1°, θ_min_ = 3.1°
0.60° ω and 0.6° φ scans	*h* = −17→17
Absorption correction: multi-scan (*SADABS*; Bruker, 2012)	*k* = −10→10
*T*_min_ = 0.529, *T*_max_ = 0.662	*l* = −18→17
30677 measured reflections	

### Refinement

**Table:**

Refinement on *F*^2^	Primary atom site location: structure-invariant direct methods
Least-squares matrix: full	Hydrogen site location: inferred from neighbouring sites
*R*[*F*^2^ > 2σ(*F*^2^)] = 0.051	H-atom parameters constrained
*wR*(*F*^2^) = 0.131	*w* = 1/[σ^2^(*F*_o_^2^) + (0.060*P*)^2^ + 2.340*P*] where *P* = (*F*_o_^2^ + 2*F*_c_^2^)/3
*S* = 1.03	(Δ/σ)_max_ < 0.001
3602 reflections	Δρ_max_ = 0.33 e Å^−^^3^
255 parameters	Δρ_min_ = −0.38 e Å^−^^3^
0 restraints	

### Special details

**Table:**

Experimental. SADABS-2012/1 (Bruker, 2012) was used for absorption correction. wR2(int) was 0.0782 before and 0.0582 after correction. The Ratio of minimum to maximum transmission is 0.8001. The λ/2 correction factor is 0.0015.
Geometry. All e.s.d.'s (except the e.s.d. in the dihedral angle between two l.s. planes) are estimated using the full covariance matrix. The cell e.s.d.'s are taken into account individually in the estimation of e.s.d.'s in distances, angles and torsion angles; correlations between e.s.d.'s in cell parameters are only used when they are defined by crystal symmetry. An approximate (isotropic) treatment of cell e.s.d.'s is used for estimating e.s.d.'s involving l.s. planes.

### Fractional atomic coordinates and isotropic or equivalent isotropic displacement parameters (Å^2^)

**Table:**

	*x*	*y*	*z*	*U*_iso_*/*U*_eq_	Occ. (<1)
Mn1	0.50071 (8)	0.53650 (7)	0.00428 (7)	0.0127 (2)	0.5
N1	0.5205 (3)	0.7069 (5)	0.0218 (3)	0.0192 (8)	0.5
N2	0.37815 (13)	0.5252 (2)	0.05324 (12)	0.0169 (4)	
N3	0.56288 (13)	0.4454 (2)	0.11560 (12)	0.0163 (4)	
C1	0.29422 (16)	0.5754 (3)	0.01319 (15)	0.0181 (5)	
C2	0.22512 (17)	0.5934 (3)	0.07588 (15)	0.0215 (5)	
H2	0.1624	0.6292	0.0649	0.026*	
C3	0.26662 (17)	0.5498 (3)	0.15313 (15)	0.0211 (5)	
H3	0.2382	0.5478	0.2068	0.025*	
C4	0.36146 (17)	0.5071 (3)	0.13943 (15)	0.0180 (5)	
C5	0.42756 (16)	0.4624 (3)	0.20622 (15)	0.0171 (5)	
C6	0.39372 (16)	0.4411 (3)	0.29439 (15)	0.0174 (5)	
C7	0.33021 (16)	0.3227 (3)	0.30958 (15)	0.0204 (5)	
H7	0.3109	0.2531	0.2641	0.024*	
C8	0.29492 (18)	0.3051 (3)	0.39015 (16)	0.0251 (5)	
H8	0.2514	0.2242	0.3989	0.030*	
C9	0.32215 (19)	0.4042 (3)	0.45822 (16)	0.0274 (6)	
C10	0.38655 (18)	0.5215 (3)	0.44361 (16)	0.0234 (5)	
H10	0.4064	0.5900	0.4895	0.028*	
C11	0.42188 (17)	0.5396 (3)	0.36346 (15)	0.0196 (5)	
H11	0.4659	0.6200	0.3551	0.023*	
C12	0.2833 (2)	0.3839 (4)	0.54568 (18)	0.0408 (7)	
H12A	0.2360	0.3011	0.5423	0.061*	
H12B	0.2542	0.4811	0.5628	0.061*	
H12C	0.3344	0.3562	0.5887	0.061*	
C13	0.52194 (16)	0.4379 (3)	0.19367 (14)	0.0172 (5)	
C14	0.59120 (16)	0.3965 (3)	0.26207 (15)	0.0196 (5)	
H14	0.5806	0.3821	0.3215	0.024*	
C15	0.67421 (16)	0.3820 (3)	0.22588 (15)	0.0193 (5)	
H15	0.7333	0.3580	0.2552	0.023*	
C16	0.65615 (16)	0.4097 (2)	0.13484 (15)	0.0168 (5)	
C17	0.72421 (16)	0.3948 (2)	0.07477 (15)	0.0167 (5)	
C18	0.82071 (16)	0.3429 (3)	0.10650 (14)	0.0185 (5)	
C19	0.83537 (17)	0.1947 (3)	0.14103 (15)	0.0222 (5)	
H19	0.7837	0.1260	0.1443	0.027*	
C20	0.92477 (18)	0.1470 (3)	0.17059 (16)	0.0277 (6)	
H20	0.9332	0.0455	0.1938	0.033*	
C21	1.00228 (17)	0.2432 (3)	0.16720 (16)	0.0287 (6)	
C22	0.98775 (17)	0.3902 (3)	0.13146 (16)	0.0280 (6)	
H22	1.0398	0.4579	0.1275	0.034*	
C23	0.89872 (17)	0.4393 (3)	0.10163 (16)	0.0240 (5)	
H23	0.8907	0.5402	0.0775	0.029*	
C24	1.09940 (19)	0.1923 (4)	0.20193 (19)	0.0436 (8)	
H24A	1.1074	0.2136	0.2643	0.065*	
H24B	1.1469	0.2496	0.1721	0.065*	
H24C	1.1067	0.0809	0.1919	0.065*	

### Atomic displacement parameters (Å^2^)

**Table:**

	*U*^11^	*U*^22^	*U*^33^	*U*^12^	*U*^13^	*U*^23^
Mn1	0.0124 (3)	0.0133 (6)	0.0122 (4)	0.0005 (5)	−0.0005 (2)	0.0007 (5)
N1	0.0141 (18)	0.021 (2)	0.022 (2)	−0.0007 (15)	−0.0009 (14)	0.0023 (16)
N2	0.0201 (10)	0.0159 (9)	0.0144 (9)	0.0013 (7)	−0.0009 (7)	−0.0007 (7)
N3	0.0191 (10)	0.0153 (9)	0.0143 (9)	0.0012 (7)	0.0009 (7)	0.0003 (7)
C1	0.0191 (11)	0.0153 (10)	0.0195 (12)	0.0010 (9)	−0.0003 (9)	−0.0008 (9)
C2	0.0199 (11)	0.0221 (12)	0.0224 (12)	0.0052 (9)	0.0013 (9)	0.0000 (10)
C3	0.0243 (12)	0.0212 (12)	0.0178 (12)	0.0044 (9)	0.0020 (9)	−0.0018 (9)
C4	0.0220 (12)	0.0160 (11)	0.0162 (11)	0.0022 (9)	0.0014 (9)	−0.0013 (9)
C5	0.0225 (12)	0.0121 (10)	0.0167 (11)	−0.0001 (8)	0.0006 (9)	−0.0021 (9)
C6	0.0191 (11)	0.0162 (11)	0.0167 (11)	0.0047 (9)	−0.0014 (8)	0.0009 (9)
C7	0.0249 (12)	0.0166 (11)	0.0192 (12)	0.0010 (9)	−0.0012 (9)	−0.0009 (9)
C8	0.0278 (13)	0.0222 (12)	0.0255 (13)	−0.0043 (10)	0.0037 (10)	0.0040 (10)
C9	0.0350 (14)	0.0304 (14)	0.0173 (12)	−0.0004 (11)	0.0056 (10)	0.0040 (10)
C10	0.0286 (13)	0.0240 (12)	0.0169 (12)	0.0027 (10)	−0.0022 (9)	−0.0020 (10)
C11	0.0221 (12)	0.0177 (11)	0.0183 (11)	0.0007 (9)	−0.0026 (9)	−0.0013 (9)
C12	0.058 (2)	0.0450 (18)	0.0214 (14)	−0.0083 (15)	0.0135 (13)	0.0014 (13)
C13	0.0230 (12)	0.0140 (10)	0.0144 (11)	0.0014 (9)	0.0001 (9)	0.0001 (9)
C14	0.0244 (12)	0.0207 (11)	0.0134 (11)	0.0037 (9)	−0.0006 (9)	−0.0004 (9)
C15	0.0223 (12)	0.0181 (11)	0.0171 (11)	0.0026 (9)	−0.0019 (9)	0.0021 (9)
C16	0.0185 (11)	0.0124 (10)	0.0191 (11)	0.0002 (8)	−0.0024 (8)	−0.0005 (9)
C17	0.0188 (11)	0.0129 (10)	0.0181 (11)	−0.0016 (8)	−0.0003 (8)	0.0008 (9)
C18	0.0179 (11)	0.0235 (12)	0.0140 (11)	0.0003 (9)	−0.0003 (8)	0.0000 (9)
C19	0.0209 (12)	0.0253 (13)	0.0205 (12)	0.0010 (10)	0.0009 (9)	0.0035 (10)
C20	0.0259 (13)	0.0354 (14)	0.0218 (12)	0.0081 (11)	0.0021 (10)	0.0091 (11)
C21	0.0177 (12)	0.0503 (17)	0.0182 (12)	0.0049 (11)	0.0014 (9)	0.0042 (12)
C22	0.0180 (12)	0.0424 (16)	0.0235 (13)	−0.0060 (11)	0.0014 (9)	0.0021 (12)
C23	0.0230 (12)	0.0273 (13)	0.0217 (12)	−0.0025 (10)	0.0012 (9)	0.0028 (10)
C24	0.0215 (14)	0.074 (2)	0.0354 (16)	0.0090 (14)	0.0013 (11)	0.0160 (16)

### Geometric parameters (Å, º)

**Table:**

Mn1—N1	1.516 (4)	C10—H10	0.9500
Mn1—N2^i^	2.070 (2)	C10—C11	1.381 (3)
Mn1—N2	1.958 (2)	C11—H11	0.9500
Mn1—N3	2.036 (2)	C12—H12A	0.9800
Mn1—N3^i^	2.010 (2)	C12—H12B	0.9800
N1—Mn1^i^	2.154 (4)	C12—H12C	0.9800
N2—Mn1^i^	2.070 (2)	C13—C14	1.439 (3)
N2—C1	1.377 (3)	C14—H14	0.9500
N2—C4	1.380 (3)	C14—C15	1.352 (3)
N3—Mn1^i^	2.010 (2)	C15—H15	0.9500
N3—C13	1.381 (3)	C15—C16	1.433 (3)
N3—C16	1.377 (3)	C16—C17	1.398 (3)
C1—C2	1.441 (3)	C17—C1^i^	1.391 (3)
C1—C17^i^	1.391 (3)	C17—C18	1.496 (3)
C2—H2	0.9500	C18—C19	1.394 (3)
C2—C3	1.348 (3)	C18—C23	1.395 (3)
C3—H3	0.9500	C19—H19	0.9500
C3—C4	1.432 (3)	C19—C20	1.385 (3)
C4—C5	1.401 (3)	C20—H20	0.9500
C5—C6	1.491 (3)	C20—C21	1.386 (4)
C5—C13	1.390 (3)	C21—C22	1.392 (4)
C6—C7	1.396 (3)	C21—C24	1.513 (3)
C6—C11	1.401 (3)	C22—H22	0.9500
C7—H7	0.9500	C22—C23	1.385 (4)
C7—C8	1.386 (3)	C23—H23	0.9500
C8—H8	0.9500	C24—H24A	0.9800
C8—C9	1.389 (4)	C24—H24B	0.9800
C9—C10	1.396 (4)	C24—H24C	0.9800
C9—C12	1.509 (3)		
			
N1—Mn1—N2	98.00 (16)	C11—C10—H10	119.5
N1—Mn1—N2^i^	100.09 (16)	C6—C11—H11	119.6
N1—Mn1—N3	99.09 (16)	C10—C11—C6	120.9 (2)
N1—Mn1—N3^i^	98.97 (16)	C10—C11—H11	119.6
N2—Mn1—N2^i^	161.90 (4)	C9—C12—H12A	109.5
N2—Mn1—N3^i^	90.30 (9)	C9—C12—H12B	109.5
N2—Mn1—N3	89.97 (9)	C9—C12—H12C	109.5
N3—Mn1—N2^i^	86.50 (9)	H12A—C12—H12B	109.5
N3^i^—Mn1—N2^i^	87.59 (9)	H12A—C12—H12C	109.5
N3^i^—Mn1—N3	161.72 (4)	H12B—C12—H12C	109.5
C1—N2—Mn1^i^	127.80 (16)	N3—C13—C5	126.2 (2)
C1—N2—Mn1	125.57 (16)	N3—C13—C14	110.1 (2)
C1—N2—C4	105.34 (19)	C5—C13—C14	123.7 (2)
C4—N2—Mn1^i^	126.48 (16)	C13—C14—H14	126.5
C4—N2—Mn1	126.96 (16)	C15—C14—C13	107.1 (2)
C13—N3—Mn1	124.89 (15)	C15—C14—H14	126.5
C13—N3—Mn1^i^	128.27 (16)	C14—C15—H15	126.5
C16—N3—Mn1	128.56 (15)	C14—C15—C16	107.0 (2)
C16—N3—Mn1^i^	125.49 (15)	C16—C15—H15	126.5
C16—N3—C13	105.32 (18)	N3—C16—C15	110.56 (19)
N2—C1—C2	110.2 (2)	N3—C16—C17	125.7 (2)
N2—C1—C17^i^	126.5 (2)	C17—C16—C15	123.6 (2)
C17^i^—C1—C2	123.3 (2)	C1^i^—C17—C16	122.9 (2)
C1—C2—H2	126.6	C1^i^—C17—C18	118.7 (2)
C3—C2—C1	106.8 (2)	C16—C17—C18	118.5 (2)
C3—C2—H2	126.6	C19—C18—C17	120.6 (2)
C2—C3—H3	126.3	C19—C18—C23	117.9 (2)
C2—C3—C4	107.4 (2)	C23—C18—C17	121.5 (2)
C4—C3—H3	126.3	C18—C19—H19	119.8
N2—C4—C3	110.2 (2)	C20—C19—C18	120.5 (2)
N2—C4—C5	126.1 (2)	C20—C19—H19	119.8
C5—C4—C3	123.6 (2)	C19—C20—H20	119.1
C4—C5—C6	117.6 (2)	C19—C20—C21	121.8 (2)
C13—C5—C4	123.0 (2)	C21—C20—H20	119.1
C13—C5—C6	119.4 (2)	C20—C21—C22	117.6 (2)
C7—C6—C5	120.1 (2)	C20—C21—C24	121.7 (3)
C7—C6—C11	117.9 (2)	C22—C21—C24	120.7 (3)
C11—C6—C5	121.9 (2)	C21—C22—H22	119.4
C6—C7—H7	119.5	C23—C22—C21	121.1 (2)
C8—C7—C6	120.9 (2)	C23—C22—H22	119.4
C8—C7—H7	119.5	C18—C23—H23	119.5
C7—C8—H8	119.5	C22—C23—C18	121.0 (2)
C7—C8—C9	121.0 (2)	C22—C23—H23	119.5
C9—C8—H8	119.5	C21—C24—H24A	109.5
C8—C9—C10	118.3 (2)	C21—C24—H24B	109.5
C8—C9—C12	120.5 (2)	C21—C24—H24C	109.5
C10—C9—C12	121.2 (2)	H24A—C24—H24B	109.5
C9—C10—H10	119.5	H24A—C24—H24C	109.5
C11—C10—C9	121.0 (2)	H24B—C24—H24C	109.5
			
Mn1^i^—N2—C1—C2	175.04 (15)	C5—C6—C7—C8	−177.2 (2)
Mn1—N2—C1—C2	−162.51 (16)	C5—C6—C11—C10	177.3 (2)
Mn1^i^—N2—C1—C17^i^	−4.7 (3)	C5—C13—C14—C15	179.8 (2)
Mn1—N2—C1—C17^i^	17.7 (3)	C6—C5—C13—N3	176.6 (2)
Mn1^i^—N2—C4—C3	−174.60 (15)	C6—C5—C13—C14	−1.8 (3)
Mn1—N2—C4—C3	162.78 (16)	C6—C7—C8—C9	−0.4 (4)
Mn1—N2—C4—C5	−13.7 (3)	C7—C6—C11—C10	−1.1 (3)
Mn1^i^—N2—C4—C5	8.9 (3)	C7—C8—C9—C10	−0.4 (4)
Mn1^i^—N3—C13—C5	−9.4 (3)	C7—C8—C9—C12	−180.0 (3)
Mn1—N3—C13—C5	13.1 (3)	C8—C9—C10—C11	0.4 (4)
Mn1^i^—N3—C13—C14	169.23 (15)	C9—C10—C11—C6	0.3 (4)
Mn1—N3—C13—C14	−168.34 (15)	C11—C6—C7—C8	1.1 (3)
Mn1^i^—N3—C16—C15	−170.66 (15)	C12—C9—C10—C11	−180.0 (3)
Mn1—N3—C16—C15	166.71 (15)	C13—N3—C16—C15	−0.9 (2)
Mn1—N3—C16—C17	−16.1 (3)	C13—N3—C16—C17	176.3 (2)
Mn1^i^—N3—C16—C17	6.5 (3)	C13—C5—C6—C7	−114.5 (2)
N2—C1—C2—C3	−1.7 (3)	C13—C5—C6—C11	67.2 (3)
N2—C4—C5—C6	−176.6 (2)	C13—C14—C15—C16	−1.7 (3)
N2—C4—C5—C13	3.4 (4)	C14—C15—C16—N3	1.7 (3)
N3—C13—C14—C15	1.2 (3)	C14—C15—C16—C17	−175.6 (2)
N3—C16—C17—C1^i^	4.2 (4)	C15—C16—C17—C1^i^	−179.0 (2)
N3—C16—C17—C18	−175.2 (2)	C15—C16—C17—C18	1.7 (3)
C1—N2—C4—C3	−1.2 (2)	C16—N3—C13—C5	−178.7 (2)
C1—N2—C4—C5	−177.7 (2)	C16—N3—C13—C14	−0.1 (2)
C1—C2—C3—C4	0.9 (3)	C16—C17—C18—C19	65.0 (3)
C1^i^—C17—C18—C19	−114.4 (2)	C16—C17—C18—C23	−115.9 (3)
C1^i^—C17—C18—C23	64.8 (3)	C17^i^—C1—C2—C3	178.0 (2)
C2—C3—C4—N2	0.2 (3)	C17—C18—C19—C20	−179.8 (2)
C2—C3—C4—C5	176.8 (2)	C17—C18—C23—C22	179.8 (2)
C3—C4—C5—C6	7.3 (3)	C18—C19—C20—C21	0.1 (4)
C3—C4—C5—C13	−172.6 (2)	C19—C18—C23—C22	−1.0 (4)
C4—N2—C1—C2	1.8 (2)	C19—C20—C21—C22	−1.1 (4)
C4—N2—C1—C17^i^	−178.0 (2)	C19—C20—C21—C24	178.2 (2)
C4—C5—C6—C7	65.5 (3)	C20—C21—C22—C23	1.0 (4)
C4—C5—C6—C11	−112.8 (3)	C21—C22—C23—C18	0.0 (4)
C4—C5—C13—N3	−3.5 (4)	C23—C18—C19—C20	0.9 (3)
C4—C5—C13—C14	178.1 (2)	C24—C21—C22—C23	−178.2 (2)

Symmetry code: (i) −*x*+1, −*y*+1, −*z*.

### Hydrogen-bond geometry (Å, º)

Cg1 and Cg2 are the centroids of the N3/C13–C16 and C6–C11 rings, respectively.

**Table:**

*D*—H···*A*	*D*—H	H···*A*	*D*···*A*	*D*—H···*A*
C10—H10···N1^ii^	0.95	2.42	3.203 (5)	140
C11—H11···*Cg*1^iii^	0.95	2.77	3.332 (3)	119
C19—H19···*Cg*2^iv^	0.95	2.68	3.619 (3)	170

Symmetry codes: (ii) *x*, −*y*+3/2, *z*+1/2; (iii) −*x*+1, *y*+1/2, −*z*+1/2; (iv) −*x*+1, *y*−1/2, −*z*+1/2.
