# Supplementary figures and images for: Crystal structure of nitrido[5,10,15,20-tetra­kis(4-methylphenyl)­porphyrinato]­manganese(V)
Source: Acta Crystallogr Sect E Struct Rep Online. 2014 Sep 24;70(Pt 10):242–5. doi: 10.1107/S1600536814020558 (PMC4257217; doi:10.1107/S1600536814020558)

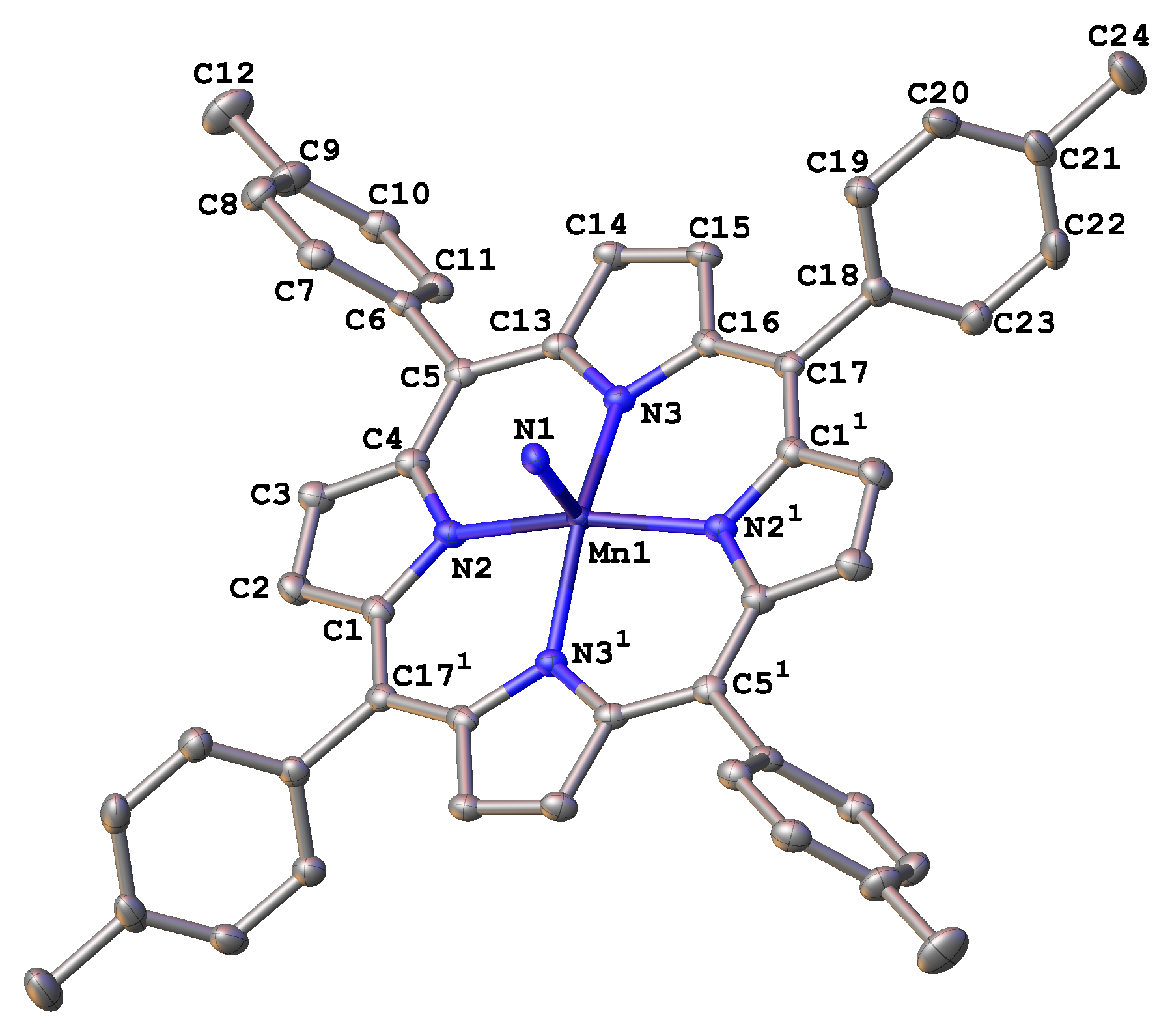

Supplement: Supplementary file 3 [file e-70-00242-Isup3.png]
